# Assessment of *Fusarium* Infection and Mycotoxin Contamination of Wheat Kernels and Flour Using Hyperspectral Imaging

**DOI:** 10.3390/toxins11100556

**Published:** 2019-09-21

**Authors:** Elias Alisaac, Jan Behmann, Anna Rathgeb, Petr Karlovsky, Heinz-Wilhelm Dehne, Anne-Katrin Mahlein

**Affiliations:** 1 Institute of Crop Science and Resource Conservation (INRES), Plant Diseases and Plant Protection, University of Bonn, Nussallee 9, 53115 Bonn, Germany; jbehmann@uni-bonn.de (J.B.); hw-dehne@uni-bonn.de (H.-W.D.); 2 Molecular Phytopathology and Mycotoxin Research, University of Goettingen, Grisebachstraße 6, 37077 Goettingen, Germany; anna.rathgeb@agr.uni-goettingen.de (A.R.); pkarlov@gwdg.de (P.K.); 3 Institute of Sugar Beet Research (IfZ), Holtenser Landstraße 77, 37079 Goettingen, Germany; Mahlein@ifz-goettingen.de

**Keywords:** *Triticum aestivum*, wheat scab, *Fusarium graminearum*, *Fusarium culmorum*, *Fusarium poae*, non-invasive sensors, fungal DNA, mycotoxins, qPCR, LC-MS/MS

## Abstract

*Fusarium* head blight (FHB) epidemics in wheat and contamination with *Fusarium* mycotoxins has become an increasing problem over the last decades. This prompted the need for non-invasive and non-destructive techniques to screen cereal grains for *Fusarium* infection, which is usually accompanied by mycotoxin contamination. This study tested the potential of hyperspectral imaging to monitor the infection of wheat kernels and flour with three *Fusarium* species. Kernels of two wheat varieties inoculated at anthesis with *F. graminearum, F. culmorum*, and *F. poae* were investigated. Hyperspectral images of kernels and flour were taken in the visible-near infrared (VIS-NIR) (400–1000 nm) and short-wave infrared (SWIR) (1000–2500 nm) ranges. The fungal DNA and mycotoxin contents were quantified. Spectral reflectance of *Fusarium*-damaged kernels (FDK) was significantly higher than non-inoculated ones. In contrast, spectral reflectance of flour from non-inoculated kernels was higher than that of FDK in the VIS and lower in the NIR and SWIR ranges. Spectral reflectance of kernels was positively correlated with fungal DNA and deoxynivalenol (DON) contents. In the case of the flour, this correlation exceeded *r* = −0.80 in the VIS range. Remarkable peaks of correlation appeared at 1193, 1231, 1446 to 1465, and 1742 to 2500 nm in the SWIR range.

## 1. Introduction

Changes in agricultural practices in particular intensification of maize production and the wide use of reduced tillage have resulted in an increased frequency of *Fusarium* head blight (FHB) epidemics worldwide [1]. Recent studies have demonstrated that the main causal agents, *Fusarium graminearum* and *F. culmorum* chemotypes, tend to show geographic specificity in the main wheat production areas [2,3,4,5]. In Europe, the predominant *F. graminearum* chemotype is 15-acetyl-deoxynivalenol (15-ADON) whereas the predominant *F. culmorum* chemotype is 3-acetyl-deoxynivalenol (3-ADON) [5]. However, drastic changes in *Fusarium* chemotypes were observed in some cereal-producing countries worldwide. For example, in Argentina, an increase in 3-ADON chemotypes was observed for four years (2001–2004). However, in Uruguay, this increase was shown in 15-ADON [6,7]. Ward et al. [8] reported a shift from the 15-ADON chemotype to the 3-ADON chemotype in Canada between 1998 and 2004. In North America, new strains of *F. graminearum* were isolated and these isolates showed type A trichothecene production, which is new for *F. graminearum* [9,10]. These results show the high ability of *Fusarium* species to adapt to variety resistance. Therefore, new varieties will be needed in the future with resistance to a wide range of *Fusarium* species and chemotypes. The first step to achieve these varieties is to screen a large number of wheat entries against FHB. For a better understanding of the host–pathogen interaction within the screening process, it should include not only disease symptoms but also the levels of fungal DNA and mycotoxins in the kernels.

The standard method for quantifying fungal DNA in infected kernels is by quantitative real-time polymerase chain reaction (qPCR) [11], whereas the methods used to detect mycotoxin contents include immunochemical methods and analytical chemistry techniques [12,13]. However, these methods are laborious, time-consuming, and destructive. Therefore, there is a need for a fast, inexpensive, and reliable non-destructive method to pre-screen wheat kernels for FHB infection and mycotoxin contamination.

Recently, hyperspectral imaging has been introduced to identify and classify damage caused by different diseases and pests on cereal kernels. Del Fiore et al. [14] used hyperspectral imaging in the visible-near infrared (VIS-NIR) (400–1000 nm) range for early identification of toxigenic fungi, i.e., *Aspergillus* spp. and *Fusarium* spp., on commercial maize kernels. In another study, hyperspectral images in the VIS-NIR range were taken for maize kernels artificially contaminated with aflatoxin B1 (AFB1) [15]. Principal component analysis (PCA) was then combined with a stepwise factorial discriminant analysis (FDA) to discriminate control samples from samples artificially contaminated with different concentrations of AFB1. The authors were able to identify kernels contaminated with concentrations as low as 10 µg/kg of AFB1 [15]. Similar results were achieved by Kimuli et al. [16], who used hyperspectral imaging in the short-wave infrared range (SWIR) (1000–2500 nm) to classify AFB1 contamination in maize kernels by combining PCA with the Mahalanobis distance classifier. Additionally, AFB1 contents have been identified and classified in *Aspergillus flavus*-infected maize kernels by applying the support vector machine (SVM) classification approach on the PCA of the mean spectra of a single kernel in the SWIR [17].

Singh et al. [18,19] used hyperspectral imaging in the NIR from 1000 to 1600 nm for the detection of insect-damaged kernels of wheat during storage. They classified healthy and insect-damaged kernels with an accuracy of 85% to 100% using different classification methods.

Peiris et al. [20] applied NIR spectroscopy from 1000 to 2500 nm to differentiate between sound and *Fusarium*-damaged kernels (FDK) contaminated with different deoxynivalenol (DON) levels. They compared the spectral reflectance of the wheat kernels with that of the serial dilutions of pure DON in acetonitrile. Subsequently, commercial application of NIR spectroscopy was investigated to predict DON levels in a single wheat kernel and to sort sound kernels from FDK [21,22]. On the bulk scale, it was possible to differentiate between sample lots with different percentages of FDK using a combination of the spectral reflectance from 350 to 2500 nm and partial least square regression with an accuracy of 100% [23]. Using the mean spectral reflectance of a single wheat kernel in VIS-NIR from 400 to 1000 and 1000 to 1700 nm and linear discriminant analysis (LDA), it was possible to discriminate FKD from sound ones with an accuracy of 95% [24,25]. Dowell et al. [26] used VIS-NIR from 400 to 1700 nm spectroscopy in combination with a partial least squares (PLS) regression model to predict DON and ergosterol in single wheat kernels. The same model was used in the VIS-NIR range to detect FDK in Canadian wheat, with an overall accuracy of 90% [27]. In the VIS range, RGB images or green and red light produced from high-power pulsed light emitting diode (LED) were used in combination with LDA to classify FDK with an accuracy of >85% [28,29]. Barbedo et al. [30] developed an algorithm for automatic detection of FDK in wheat kernels based on hyperspectral imaging in the spectral range 528–1785 nm.

All the above studies were performed to identify *Fusarium* infection in wheat kernels using one species of *Fusarium* taking the DON content into account. To expand that knowledge base, the present study was designed to investigate the feasibility of hyperspectral imaging to screen the infection of different *Fusarium* species with regard to fungal DNA and different mycotoxin levels in wheat kernels and produced flour.

## 2. Results

### 2.1. Effect of Fusarium Infection on the Spectral Signature of the Kernels

**Figure 1:**
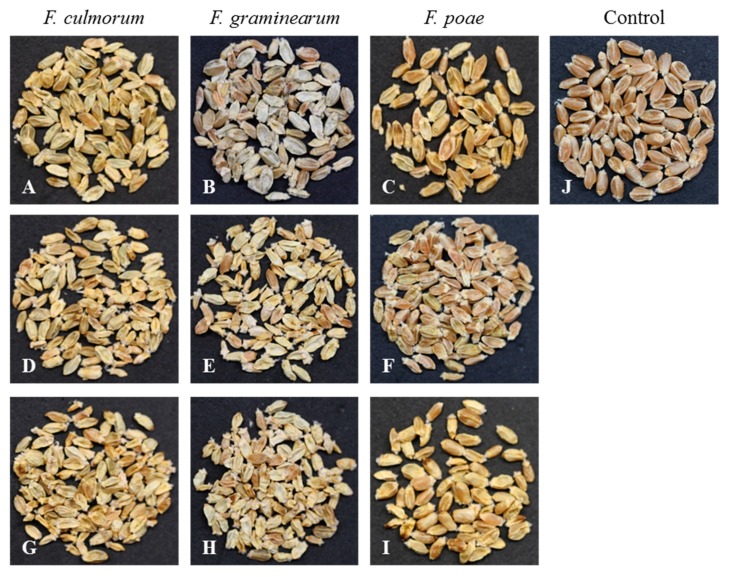
Summer wheat kernels cv. ‘Sonett’ inoculated with different spore densities (**A**–**C**): 1 × 10^4^ spore/mL; (**D**–**F**): 2.5 × 10^5^ spore/mL; (**G**–**I**): 1 × 10^6^ spore/mL; (**J**): water treatment of three *Fusarium* species (**A**, **D**, and **G**: *F. culmorum*; **B**, **E**, and **H**: *F. graminearum*; **C**, **F**, and **I**: *F. poae*; **J**: Control).

**Figure 2:**
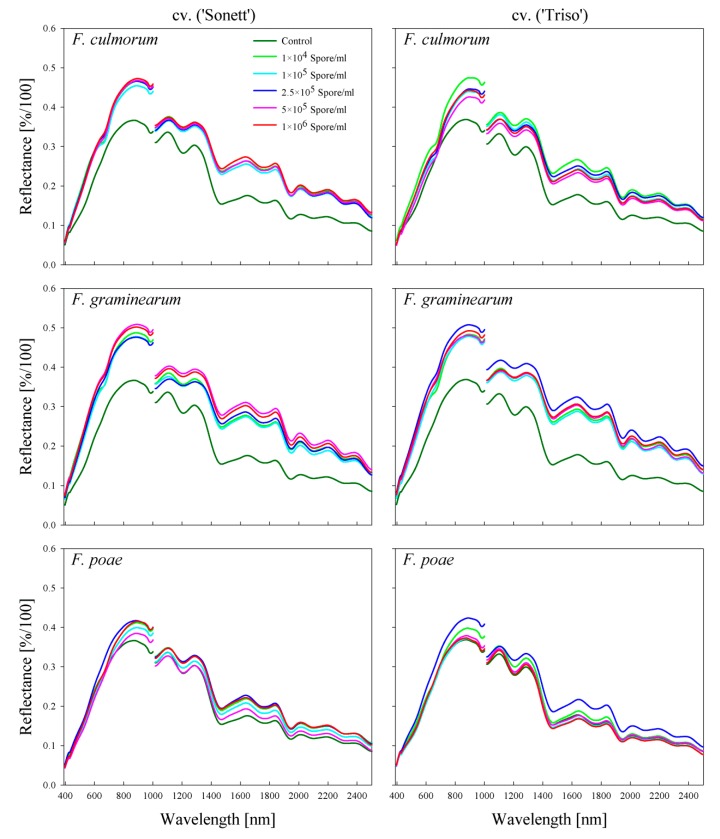
Spectral signature of wheat kernels cv. ‘Sonett’ and ‘Triso’ inoculated with different spore densities of three *Fusarium* species (*F. culmorum, F. graminearum*, and *F. poae*).

*Fusarium*-infected kernels of both varieties showed sever symptom for all investigated *Fusarium* species and all spore densities (Figure 1). Non-inoculated kernels of both varieties had the same spectral signature patterns in the VIS-NIR and SWIR ranges (Figure 2). In contrast, *Fusarium*-infected kernels showed higher spectral reflectance along the whole spectrum compared with non-inoculated kernels.

The spectral signature of *Fusarium*-infected kernels differed according to the wheat variety, *Fusarium* species, and spore density of the inoculum (Figure 2). *F. culmorum*-infected kernels of ‘Sonnet’ from all spore densities showed identical spectral reflectance from 400 to 630 nm. Small differences among treatments appeared in the spectral range from 630 to 700 nm and from 750 to 1000 nm. In the SWIR range, these differences were pronounced from 1460 to 1850 nm (Figure 2). More obvious differences among treatments were shown in the case of ‘Triso’. The highest reflectance of kernel samples resulted from the treatment of 1 × 10^4^ spore/mL, whereas the lowest reflectance was for the kernels from the treatment of 5 × 10^5^ spore/mL (Figure 2).

*F. graminearum* infection affected the same wavelengths that were affected by *F. culmorum* infection in the VIS-NIR range (i.e., from 400 to 630 nm and from 750 to 1000 nm), whereas the entire SWIR range was influenced by *F. graminearum* infection. The treatment of 1 × 10^5^ spore/mL resulted in the lowest reflectance of infected kernels in both varieties. The highest reflectance appeared in the treatment of 5 × 10^5^ spore/mL in ‘Sonett’ and 2.5 × 10^5^ in ‘Triso’. *F. poae* infection influenced only the NIR and the SWIR ranges. The highest reflectance was found for the treatment of 2.5 × 10^5^ spore/mL in both varieties, and the lowest levels were with the treatment of 5 × 10^5^ in ‘Sonett’ and 1 × 10^6^ in ‘Triso’ (Figure 2).

### 2.2. Comparison between the Spectral Signatures of the Kernels and the Produced Flour

**Figure 3:**
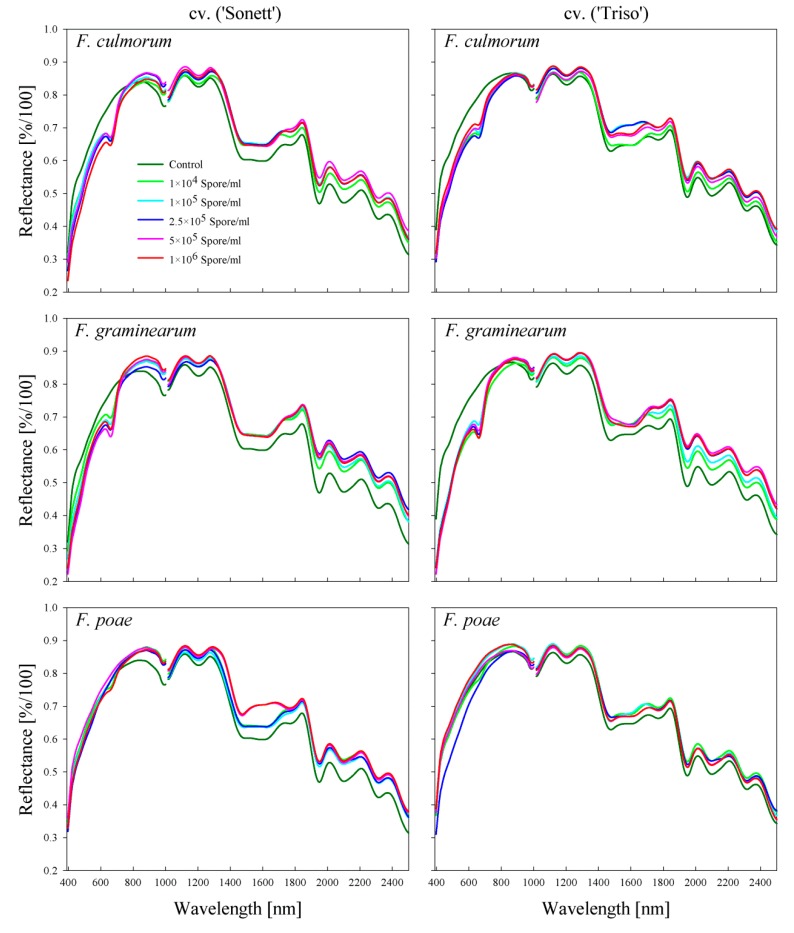
Spectral signature of the flour samples produced from wheat kernels cv. ‘Sonett’ and ‘Triso’ inoculated with different spore densities of three *Fusarium* species (*F. culmorum, F. graminearum*, and *F. poae*).

The spectral signature patterns of flour differed from those of kernels (Figure 2 and Figure 3). The spectral reflectance of non-inoculated kernels was lower than that of infected kernels along the assessed waveband range. However, in the case of flour, the spectral reflectance of non-inoculated flour was significantly higher than that of infected flour in the VIS range (Figure 3). Obvious differences appeared from 630 to 670 nm in the spectral signatures of the flour produced from *F. culmorum*- and *F. graminearum*-infected kernels (Figure 3). Flour produced from *F. poae*-infected kernels showed no distinct differences in the spectral signatures in the VIS range. The treatment with the 2.5 × 10^5^ spore/mL concentration, which resulted in the highest spectral reflectance of the infected kernels, caused the lowest spectral reflectance of the flour in both varieties (Figure 3). The spectral reflectance of the flour produced from non-inoculated kernels had the lowest values in the spectral range from 870 to 1000 nm in all treatments. In the SWIR range, the infection altered the wavelengths from 1100 to 1300 and 1950 to 2500 nm. In general, flour produced from non-inoculated kernels had lower reflectance than infected ones in the SWIR range (Figure 3).

### 2.3. Fungal DNA and Mycotoxin Content in Wheat Flour

Flour produced from non-inoculated kernels of both varieties showed neither fungal DNA nor mycotoxins for any of investigated *Fusarium* species.

**Figure 4:**
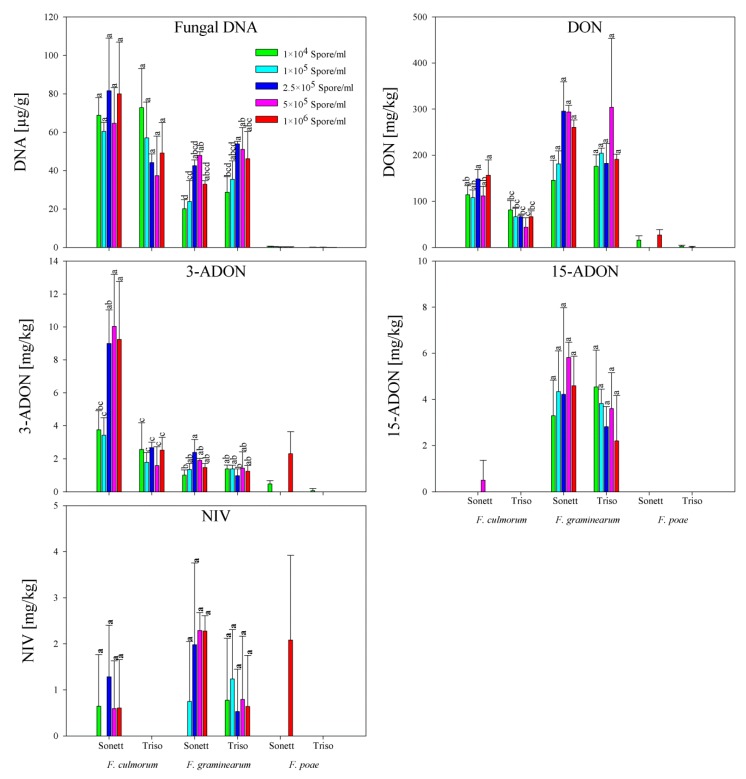
Fungal DNA and mycotoxin contents in the flour samples produced from wheat kernels cv. ‘Sonett’ and ‘Triso’ inoculated with different spore densities of three *Fusarium* species (*F. culmorum, F. graminearum*, and *F. poae*; Tukey’s test; *n* = 3). On the species scale, treatments with the same letters are not significantly different.

*F. culmorum* produced a large amount of fungal DNA in both varieties amounting to 60 to 82 and 37 to 73 µg/g in ‘Sonett’ and ‘Triso’, respectively. Additionally, both varieties showed high DON content: 108 to 157 mg/kg in ‘Sonett’ and 44 to 81 mg/kg in ‘Triso’. Fungal DNA and DON contents were not significantly different among treatments within the same variety. In addition, small amounts of 3-ADON (up to 10 and 2.7 mg/kg in ‘Sonett’ and ‘Triso’, respectively) were observed. Only in ‘Sonett’ were low levels of 15-ADON (0.5 mg/kg) and nivalenol (NIV) (up to 1.3 mg/kg) detected. The variety ‘Sonett’ showed higher levels of fungal DNA and mycotoxins than ‘Triso’ (Figure 4).

Comparing with *F. culmorum*, *F. graminearum* resulted in lower fungal DNA and 3-ADON and higher DON, 15-ADON and NIV content in all treatments. In contrast with *F. culmorum*, ‘Sonett’ showed lower fungal DNA amounts 20 to 48 µg/g comparing with ‘Triso’ 29 to 54 µg/g. DON contents ranged between 146 to 296 mg/kg in ‘Sonett’ and 176-304 mg/kg in ‘Triso’. Maximum amounts of 2.4, 4.5 and 2.3 mg/kg have been observed for 3-ADON, 15-ADON and NIV, respectively, with no significant differences between treatments (Figure 4).

In contrast to *F. culmorum* and *F. graminearum* infections, *F. poae* induced very low amounts of fungal DNA that ranged between 0.1 and 0.5 µg/g, without significant differences among treatments. Maximum concentrations of 27, 2.3, and 2.1 mg/kg for DON, 3-ADON, and NIV, respectively, were observed in ‘Sonett’. In contrast, in ‘Triso’, 2.8 and 0.1 mg/kg for DON and 3-ADON, respectively, were recorded. No 15-ADON contamination was observed in the two varieties (Figure 4).

### 2.4. Correlation between Fungal DNA and Mycotoxin Content

The data of all samples of both varieties inoculated with different spore densities was used to calculate the correlation between fungal DNA and mycotoxin content for each *Fusarium* species. Table 1 shows the correlation between fungal DNA and mycotoxins content and the correlation between different mycotoxins for each *Fusarium* species, separately. In the case of *F. poae* infection, the correlations between fungal DNA and mycotoxin contents, and individual mycotoxin content with the other mycotoxins were not significant since most of the samples showed no mycotoxin content (Table 1).

### 2.5. Correlation of Spectral Signature of Wheat Kernels to Fungal DNA and Mycotoxin Content

**Figure 5:**
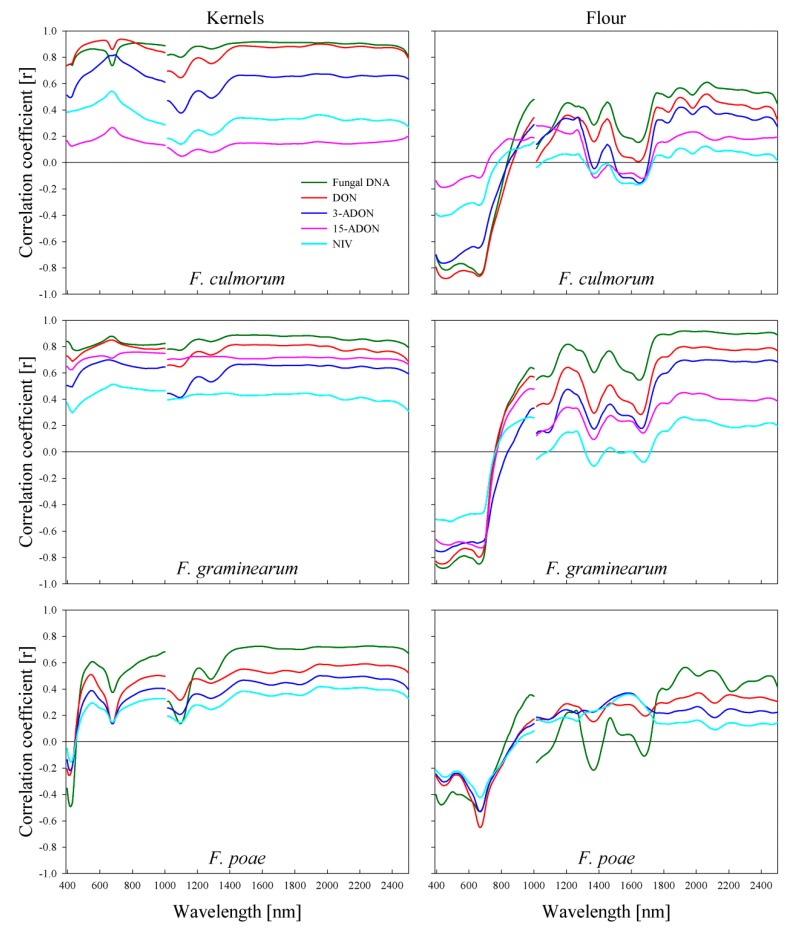
Pearson’s correlation coefficient between the spectral signature of wheat kernels cv. ‘Sonett’ and ‘Triso’ (left) inoculated with different spore densities of three *Fusarium* species and wheat flour produced from these kernels (right) in relation to fungal DNA and various mycotoxin levels.

The spectral reflectance of *F. culmorum*-infected kernels showed a high correlation of *r* > 0.80 with the fungal DNA content in the spectral range 450–652 and 700–750 nm and along the SWIR range (Figure 5). This correlation reached *r* = 0.90 in the spectral range 750–1000 nm. The correlation of the spectral reflectance with DON content showed high values of *r* > 0.80 in the spectral range 441–1000 nm. This correlation was *r* > 0.90 in the spectral ranges from 505 to 655 and 695 to 843 nm. In the SWIR range, the correlation of *r* > 0.80 was shown in the spectral ranges from 1200 to 1212 and 1345 to 2500 nm, with a peak of *r* > 0.90 in the spectral range 1906–2018 nm. A lower correlation of *r* > 0.60 occurred for the 3-ADON content in the spectral ranges from 460 to 1000 and 1377 to 2500 nm, with a remarkable peak of *r* > 0.80 in the spectral range from 640 to 712 nm.

The correlation of the spectral reflectance of *F. graminearum*-infected kernels with the fungal DNA content was *r* > 0.80 in the spectral ranges 524–1000 and 1130–2500 nm (Figure 5). Similarly, the correlation with DON content resulted in the same values in the spectral ranges 558–814 and 1377–2031 nm. The correlation of the spectral reflectance with the DON derivative contents was lower compared with the DON content. This correlation ranged between *r* = 0.60–0.70 in the spectral ranges 474–1000 and 1358–2500 nm for the 3-ADON content, and between *r* = 0.62 and 0.76 along the electromagnetic spectrum for 15-ADON. No correlation between the spectral reflectance and NIV was observed.

The spectral reflectance of *F. poae*-infected kernels showed a lower correlation with the fungal DNA content compared with *F. culmorum* and *F graminearum* (Figure 5). A correlation of *r* > 0.60 in the spectral ranges 538–572 and 828–1000 nm was proven. In the SWIR range, the correlation ranged between *r* = 0.60 and 0.73 in the spectral range 1350–2500 nm.

### 2.6. Correlation of Spectral Signature of Wheat Flour to Fungal DNA and Mycotoxin Content

The spectral reflectance of the produced flour resulted in negative correlations with the fungal DNA and mycotoxin contents in the VIS range. In addition, a positive correlation in the SWIR range and no significant correlation in the NIR range were confirmed (Figure 5).

The spectral reflectance of flour produced from *F. culmorum*-infected kernels exhibited a high negative correlation of *r* > 0.80 with the fungal DNA content in the spectral ranges 427–494 and 601–692 nm. In the SWIR range, two remarkable peaks of *r* > 0.45 appeared in the spectral ranges 1193–1231 and 1446–1465 nm. The correlation increased to *r* > 0.51 in the spectral range 1742–2500 nm and reached *r* = 0.61 in 2050 to 2075 nm (Figure 5). The correlation with DON content was higher than the correlation with the fungal DNA in the VIS range, and lower in the SWIR range. The correlation reached *r* > 0.85 in the spectral ranges 427–494 and 655–675 nm; however, the highest value of *r* = 0.52 appeared in the range 2050–2075 nm. The correlation with 3-ADON content showed decreasing values with an increasing wavelength in the VIS range and ranged between 0.70 and 0.50, with no remarkable correlation in the SWIR range (Figure 5).

The spectral reflectance of flour produced from *F. graminearum*-infected kernels had a higher correlation with fungal DNA than that of *F. culmorum*. The highest negative correlation of *r* > 0.85 appeared in the spectral ranges 397–494 and 652–670 nm. Likewise, for *F. culmorum*, the same peaks appeared in the spectral ranges 1193–1231 and 1446–1465 nm, with a higher correlation of *r* > 0.81 and *r* > 0.76, respectively, and increased to *r* > 0.86 in the spectral range 1742–2500 nm (Figure 5). The correlation with DON content showed values of *r* > 0.80 and *r* > 0.79 in the spectral ranges 397–494 and 655–675 nm, respectively. In the SWIR range, the correlation reached *r* > 0.64, *r* > 0.50, and *r* = 0.65 in the spectral ranges 1193–1231, 1446–1465, and 1742–2500 nm, respectively. In the VIS range, the correlation with 3-ADON showed a decreasing value with an increasing wavelength, with a peak of 0.76 in the spectral range 422–435 nm. While, it showed the reverse with 15-ADON, with a peak of *r* = 0.69 in the spectral range 635–700 nm. The correlation with 3-ADON reached *r* > 0.46 and *r* > 0.60 in the spectral ranges 1193–1231 and 1800–2500 nm, respectively. In contrast, no correlation was observed with 15-ADON in the SWIR range. The NIV content showed no significant correlation with the spectral reflectance of the produced flour, and the highest negative correlation of *r* = 0.53 was achieved in the spectral range 475–497 nm (Figure 5).

The spectral reflectance of flour produced from *F. poae*-infected kernels had the highest correlations with fungal DNA of *r* = 0.53 and *r* = 0.56 in the spectral ranges 664–672 and 1912–1950 nm, respectively (Figure 5).

## 3. Discussion

The symptoms of *Fusarium* infection on infected kernels result in a reduction of the thousand kernel weight, shriveling, pinkish discoloration, and chalky appearance [1,31]. Several studies showed the applicability of hyperspectral imaging for the detection of *Fusarium*-damaged kernels or spikelets [20,21,22,23,24,25,32,33]. The reflected light in the VIS range shows the tissue pigments; the NIR range represents the tissue structure, while the SWIR range characterizes the chemical compounds [34]. In the current study, the reflectance of infected kernels was higher than that of non-infected ones in the VIS-NIR range. This is attributed to the discoloration of infected kernels compared with healthy ones, and to the changes in the kernel structure due to infection. This is in accordance with previous studies that proved a lower reflectance of healthy wheat kernels compared with *Fusarium*-damaged kernels in the VIS-NIR range [23,25,27]. Former studies demonstrated an increase in the protein, starch, starch lipids, and non-starch lipids in *Fusarium*-infected kernels [35,36,37]. The reflectance of infected kernels was higher than of healthy ones in the SWIR range, which is consistent with the results of [23,24,25]. This is due to the changes in the chemical contents of the infected kernels due to *Fusarium* infection.

The quality of wheat flour depends strongly on gluten proteins [38]. *Fusarium* infection leads to substantial changes in the storage contents of wheat kernels [39], and consequently, flour appearance. Histological investigation proved high degradation in the starch granules and the protein matrix of the endosperm of *Fusarium*-infected kernels [40]. Gärtner et al. [41] showed a positive correlation (*r* = 0.93) between the disease severity and ash content (minerals like potassium and calcium salts) of *F. culmorum*-infected kernels. This explains the lower reflectance of flour from infected kernels compared with that from non-infected kernels in the current study.

The study of Hellin et al. [42] showed a high correlation between the spore density in the air above the canopy and DON concentration and disease incidence of *F. graminearum* under field conditions. In the current study, no significant differences were found between inoculum density and fungal DNA and mycotoxin concentrations in the infected kernels with all *Fusarium* species. This is due to the differences in the experimental conditions of these studies. While the study of Hellin et al. [42] was implemented under natural conditions in the field and without incubation, the current study was implemented under greenhouse conditions with incubation for 48 h after inoculation. Under these optimal conditions for the pathogens, the lowest number of spores was enough to induce infection with maximum disease severity, which could not be further increased using higher spore densities.

In this study, *F. culmorum* isolate produced DON and 3-ADON as secondary metabolites, while *F. graminearum* produced DON, 3-ADON, and 15-ADON. This is in accordance with the results that showed the ability of *F. graminearum* to produce DON, 3-ADON, and 15-ADON simultaneously [43]. Small amounts of NIV were detected in some samples. This is due to a side infection with NIV producer, since the isolates used in this study are DON producers. The results of the current study showed high correlations of *r* = 0.90, *r* = 0.70, and *r* = 0.85 between fungal DNA–DON, fungal DNA–3-ADON, and DON–3-ADON, respectively, for *F. culmorum*. In addition, correlations of *r* = 0.80, *r* = 0.51, and *r* = 0.79 between fungal DNA–DON, fungal DNA–15-ADON and DON–15-ADON, respectively, for *F. graminearum* were observed. This indicates that the major source of DON, 3-ADON, and 15-ADON in the samples resulted from the isolates used for inoculation, and not from a spontaneous infection. Moreover, it shows that these isolates produce trichothecenes in the same ratio in vitro and in vivo. In the case of *F. poae*, no significant correlation was seen because it was less effective in inducing infection in both wheat varieties. This corresponds to the results of previous studies that showed high correlations between the fungal DNA of *F. culmorum* and *F. graminearum* and the amount of DON in infected kernels of wheat and barley. In addition, a positive correlation between the fungal DNA of *F. poae* and NIV content was documented. However, on the species scale, it was shown previously that *F. poae* was less effective in term of fungal DNA and mycotoxin production [44,45,46].

The results of the current study showed higher correlations in the NIR range compared with the VIS range between the spectral reflectance of wheat kernels and the fungal DNA and DON contents for all *Fusarium* species. This is in accordance with the results of Shahin and Symons [27] that proved no specific wavelengths correlated to *Fusarium* infection in wheat kernels in the VIS-NIR ranges. The results of Delwiche et al. [25] showed that the spectral ranges 502–678 nm, 1198–1496 nm, and 1420–1560 nm were affected by *Fusarium* infection. They showed that the NIR range was superior to the VIS range to identify *Fusarium*-infected kernels. They attributed this to the transformation of the endosperm compound into fungal characteristic compounds, i.e., chitin and ergosterol. Delwiche [47] showed that the wavelengths 1182 and 1242 nm gave the best results to discriminate sound kernels from *Fusarium*-damaged kernels. The study of Delwiche and Hareland [24] showed that the spectral range 1130–1190 nm was strongly affected by *Fusarium* infection, with a peak at 1158 nm. In addition, the spectral range 1300–1450 nm was affected with a remarkable peak at 1400 nm. In the current study, the same peak of correlation between the spectral reflectance and the fungal DNA and DON contents appeared in the spectral range 1200–1212 nm. However, a higher correlation has been proven in the spectral range 1350–2500 nm for all *Fusarium* species.

In the current study, more pronounced peaks of correlations appeared between the spectral reflectance of wheat flour and fungal DNA and DON contents compared with wheat kernels. Dowell et al. [26] showed that the wavelengths of 750 and 950 nm were characteristic bands in the VIS-NIR range for predicting *Fusarium*-damaged kernels. They attributed this to the effect of *Fusarium* infection on the starch and protein content of wheat kernels, which absorb the radiation of these bands. In the current study, two pronounced peaks between the flour spectral reflectance and fungal DNA and DON contents were shown in the spectral ranges 427–494 and 655–670 nm. No correlation was found in the NIR range, which indicates the tissue structure. This is attributed to the destruction of the kernel tissue structure during grinding.

In the SWIR, Peiris et al. [20] showed that the absorption bands of different DON concentrations dissolved in acetonitrile were in 1390 to 1440 and 1880 to 1950 nm. They proved clear peaks at 1414 nm due to the –OH groups and 1906 nm due to the -C=O and R-OH groups of the DON molecule. Moreover, they proved that the bands 1195, 1208, 1365, 1445, 1700, 1905, and 2001 nm were characteristic bands for *Fusarium*-infected kernels. This is in accordance with the results of the current study, which showed two remarkable peaks of correlation between the spectral signature of the flour and fungal DNA and DON contents in the spectral ranges 1193–1231 and 1446–1465 nm, in addition to a high correlation along the spectral range 1742–2500 nm.

## 4. Conclusions

This study showed that *Fusarium* infection influences the spectral signatures of wheat kernels and flour produced from them. Correlations between the spectral data of infected kernels and corresponding flour, fungal DNA, and individual mycotoxins were documented. This shows the feasibility of hyperspectral imaging to screen wheat kernels and flour for *Fusarium* infection. Previous results showed the suitability of hyperspectral imaging to phenotype *Fusarium* head blight on wheat at the spike scale. Combined with the results of this study, hyperspectral imaging provides the breeder with an integrated tool to phenotype *Fusarium* infection on the spike scale and in harvested grain, accelerating the phenotyping process.

## 5. Materials and Methods

### 5.1. Plant Material

Two varieties of spring wheat (*Triticum aestivum* L.) differing in their susceptibility to FHB were grown under greenhouse conditions: ‘Triso’ moderately resistant from DSV, Lippstadt, Germany, and ‘Sonett’ moderately susceptible from Syngenta, Basel, Switzerland (Descriptive List of Varieties, Bundessortenamt, Germany, 2017). The growth substrate was a mixture of 1 sand:3 horizon C:6 potting substrate ED 73 (Einheitserde, Sinntal-Altengronau, Sinntal, Germany). Three kernels per pot were planted in 12 × 12 × 20 cm-sized pots. After germination, the plants were thinned to two plants per pot and the plants were supported by wooden sticks. Environmental conditions in the greenhouse were: Photoperiod of 16/8 h (day/night) obtained from supplemental artificial light (>300 µmol m^−2^ s^−1^, Philips SGR 140, Hamburg, Germany); a temperature of 20 ± 2 °C; and 50–70% relative humidity. The plants were watered when necessary.

### 5.2. Plant Pathogens and Inoculation Techniques

Isolates of three *Fusarium* species stored at −80 °C were used in this study: *Fusarium graminearum*, isolate S.19 produced 69% 15-ADON and 31% DON, and *F. culmorum*, isolate 3.37 produced 68% 3-ADON and 32% DON on rice culture. They were originally isolated from infected wheat kernels in an experimental field (Campus Klein-Altendorf, Rheinbach, Germany) in 2011 and 2004, respectively. *F. poae*, isolate EC15 was taken from the INRES (Plant Diseases and Plant Protection, University of Bonn, Germany) mycological collection. Pathogen cultivation and inoculum preparation were performed according to Alisaac et al. [32]. The spore suspension was diluted to 1 × 10^6^ spore/mL using a Fuchs–Rosenthal chamber. Spore densities of 1 × 10^4^, 1 × 10^5^, 2.5 × 10^5^, 5 × 10^5^, and 1 × 10^6^ spore/mL were derived from the stock spore suspension and used in inoculation. Each treatment consisted of one pot with two plants of each variety with more than 10 spikes per pot at the anthesis growth stage, GS 61–65 [48]. Pots were randomly distributed on the table in the middle of the greenhouse in order to guarantee the homogeneity of the surrounding environment. Spray inoculation was done as described in Alisaac et al. [32]. Control plants were mock inoculated with water. At harvest, spikes were collected and threshed manually, and the resulting kernels prepared for hyperspectral measurement (Figure 1). After acquiring the hyperspectral images of the kernels, they were ground using a lab grinder (Retsch MM 200, Haan, Germany). The produced flour was measured using the same hyperspectral imaging setup with the same settings.

### 5.3. Hyperspectral Measurements

Hyperspectral measurements were done in a darkened room. The objects were illuminated using an artificial light produced from six ASD-Pro-Lamps (Analytical Spectral Devices Inc., Boulder, CO, USA) with a 45° vertical angle to the objects. The images were acquired in the visible-near infrared (VIS-NIR) range from 400 to 1000 nm using the hyperspectral camera ImSpector V10E (Spectral Imaging Ltd., Oulu, Finland). In total, 211 hyperspectral bands were recorded using a spectral binning of 4. In the short-wave infrared (SWIR) from 1000 to 2500 nm, a SWIR-Camera (ImSpector N25E, Spectral Imaging Ltd., Oulu, Finland) was used. In total, 256 hyperspectral bands were recorded using a spectral binning of 1. A motorized line scanner (Velmex BiSlide, Velmex Inc., Bloomfield, Ontario County, NY, USA) was used to move the cameras and the illumination system over the object. The software SpectralCube (Spectral Imaging Ltd., Oulu, Finland) was applied to adjust the settings of the camera and the motorized line scanner. To attain stable measuring conditions, the cameras and the illumination system were preheated for 30 min before the measurements. For more details about the imaging system and data processing, see Alisaac et al. [32]. To acquire the hyperspectral images, a black Petri dish (Brightic, Nagymaros, Hungary) was used as a background for the kernel and flour measurements. This Petri dish has low reflectance of <5% in the spectral range of 400 to 2500 nm. For flour measurement, black rings of a 1 mm height were filled with the flour in order to obtain a homogeneous topography for the flour surface. Four images were made to calculate the reflectance of the object sample: (i) A white reference image using a white barium-sulfate bar (Spectral Imaging Ltd., Oulu, Finland), which reflects ~99% of the light; (ii) a dark current image by closing the shutter of the camera at the same exposure time of the white reference; (iii) the image of the object sample; and (iv) a second dark current image at the same exposure time of the object sample. The open source software HSVaP (“Hyperspectral Visualization and Processing”) was used for masking and visualizing the image data (available at https://github.com/janBehmann/HSVAP). The mean spectral reflectance was calculated using MATLAB 2013a (MathWorks, Natick, MA, USA).

### 5.4. DNA Extraction and Fungal DNA Quantification

Total DNA was extracted from 20 mg of flour according to a cetyltrimethylammonium bromide method and precipitated with polyethylene glycol [49]. The pellets were washed twice with 80% (*v*/*v*) ethanol, dried in vacuum at 30 °C, dissolved in 50 µL TE-buffer (10 mM Tris, 1 mM EDTA, pH 8.0), and diluted 100-fold in water for qPCR. Three-fold dilution series of pure fungal DNA from 0.3 to 100 pg per reaction were used as standards. A real-time PCR thermocycler CFX 384 (Biorad, Rüdigheim, Germany) was used for fungal DNA quantification using the primers listed in Table 2. The components of the reaction mixture were: Taq Polymerase with ThermoPol Buffer (20 mM Tris-HCl, 10 mM (NH_4_)_2_SO_4_, 10 mM KCl, 2 mM MgSO_4_, 0.1% Triton-X-100, pH 8.8 at 25 °C); 0.15 mM of each dNTP; 2.5 mM MgCl_2_ (Bioline, Lückenwalde, Germany); 0.3 μm of each primer; and SYBR Green I (Invitrogen, Karlsruhe, Germany). The PCR started with initial denaturation at 95.0 °C for 2 min, followed by 35 cycles according to Table 2. The final elongation was done at 68.0 °C for 5 min. Melting curves were generated after amplification by heating PCR products to 95.0 °C for 1 min, cooling to 55.0 °C for 1 min, then tardily raising the temperature 0.5 °C/10 s according to Table 2. The fluorescence was measured continuously. Because of degradation of wheat DNA in infected kernels, the amount of fungal DNA per gram of dry flour rather than the ratio of fungal to wheat DNA was used as a measure of fungal colonization.

### 5.5. Mycotoxin Extraction and Quantification

Mycotoxins were extracted from 350 mg of flour per treatment in 3.5 mL of acetonitrile-water (84:16, *v*/*v*). The mixture was vortexed, shaken overnight, and centrifuged at 4500 min^−1^. One mL of the supernatant was transferred to a 2-mL Eppendorf tube and dried at 40 °C. The dry residue was re-suspended in 1 mL of methanol-1% formic acid in water (25:75, *v*/*v*), and sonicated in an ultrasonic bath until completely dissolved. Blank (control) samples of wheat flour were prepared in the same way. The following mycotoxin standards were used (Table 3): Nivalenol (NIV), deoxynivalenol (DON), 3-acetyl-deoxynivalenol (3-ADON) and 15-acetyl-deoxynivalenol (15-ADON), HT-2 toxin, T-2 toxin, zearalenone (ZEA), enniatin A1, and enniatin B1.

An Agilent (Waldbronn, Germany) 1290 Infinity II HPLC system linked to an Agilent 6460 Triple Quad was employed for toxin quantification. Separation was carried out on an Agilent Zorbax Eclipse C18 column with a 1.8-µm particle size and 100 × 2.1 mm. Details concerning the mass transition, recoveries, and the limits of detection and quantification can be found in Table 3.

### 5.6. Statistical Analysis

All Pearson’s correlation coefficients and statistical analysis were done using the open source software RStudio. For significant statistical differences in the fungal DNA and mycotoxins contents, standard analysis of variance (ANOVA) was applied on the data. This analysis was followed by Tukey’s honest significance test with a significance level of *p* ≤ 0.05, *n* = 3 (“agricolae" package in RStudio).

## Figures and Tables

**Table 1:** Pearson’s correlation coefficient between fungal DNA and concentrations of different mycotoxins in the flour samples produced from wheat kernels cv. ‘Sonett’ and ‘Triso’ inoculated with different spore densities of three *Fusarium* species (*F. culmorum, F. graminearum*, and *F. poae*).

Fungal DNA-Mycotoxin	*F. culmorum*	*F. graminearum*	*F. poae*
Fungal DNA-DON	0.90	0.80	n.s.
Fungal DNA-3-DON	0.70	n.s.	n.s.
Fungal DNA-15-DON	n.s. *	0.51	n.s.
DON-3-ADON	0.85	n.s	n.s.
DON-15-ADON	n.s.	0.79	n.s.

The correlation was calculated from the data of all samples inoculated with different spore densities of each *Fusarium* species (*n* = 30). * n.s. the correlation was not significant at *p* ≤ 0.05.

**Table 2:** Forward and reverse primers sequences; denaturation, annealing, elongation, and melting curve temperatures of the primer used to amplify specific fragments of the fungal DNA of *Fusarium culmorum*, *F. graminearum*, and *F. poae* on wheat kernels and in flour.

Pathogen	Denaturation		Annealing		Elongation		Melt Curve		Primers			
	°C	min	°C	min	°C	min	°C	°C	Primer name	Primer sequence (5′-3′)	Amplified fragment	Reference
*F. culmorum*	94.0	0:20	62.0	0:40	68.0	0:45	65.0	95.0	OPT18 F OPT18 R	GATGCCAGACCAAGACGAAG GATGCCAGACGCACTAAGAT	472 bp	Schilling et al. [49]
*F. graminearum*	94.0	0:30	61.0	0:30	68.0	0:30	55.0	95.0	Fg16N F Fg16N R	ACAGATGACAAGATTCAGGCACA TTCTTTGACATCTGTTCAACCCA	280 bp	Nicholson et al. [50]
*F. poae*	94.0	0:35	62.5	0:30	68.0	0:35	55.0	95.0	Fp82 F Fp82 R	CAAGCAAACAGGCTCTTCACC TGTTCCACCTCAGTGACAGGTT	220 bp	Parry and Nicholson [51]

**Table 3:** Mass transitions, recoveries, and the limits of detection and quantification of mycotoxins in flour samples produced from wheat kernels cv. ‘Sonett’ and ‘Triso’ inoculated with different spore densities of three *Fusarium* species (*F. culmorum, F. graminearum*, and *F. poae*).

Toxin	Obtained From	Molecular Ion	Parent Ion	Collision Energy [V]	Product Ions	LOD *	LOQ *	Recovery ***
						[mg/kg]	[mg/kg]	%
NIV	Merck	[M-H]^−^	357.1	10	311.1	0.007	0.025	89
	(Darmstadt, Germany)			10	281.1 **			
DON	Merck	[M+H]^+^	297.1	4	249.1 **	0.006	0.018	113
	(Darmstadt, Germany)			64	91.2			
3-ADON	Merck	[M+H]^+^	339.2	8	231.1 **	0.022	0.072	110
	(Darmstadt, Germany)			8	203			
15-ADON	Merck	[M+H]^+^	339.2	10	261.0 **	0.07	0.23	111
	(Darmstadt, Germany)			10	203			
HT-2	Enzo Life Sciences	[M+Na]^+^	447	17	345.1 **	0.029	0.097	96
	(Lörrach, Germany)			17	285			
T-2	Enzo Life Sciences	[M+Na]^+^	489.2	98	128.1	0.029	0.092	119
	(Lörrach, Germany)			142	115.1 **			
ZEA	Romer Labs	[M+H]^+^	319.2	12	301.1	0.098	0.322	76
	(Tulln, Austria)			12	283.0 **			
Enniatin A1	Merck	[M+H]^+^	668.4	20	228.2	0.029	0.095	68
	(Darmstadt, Germany)			20	210.2 **			
Enniatin B1	Merck	[M+H]^+^	654.4	23	228.2	0.046	0.151	80
	(Darmstadt, Germany)			23	210.2 **			

* Limit of detection (LOD) and limit of quantification (LOQ) were estimated according to a procedure suggested by an EU guidance document by spiking 10 blank samples to 1.9 µg/L of each toxin. ** Product ion used as quantifier. *** Spike level of 300 µg/L at the beginning of the extraction procedure.
